# Developing Behaviour Change Interventions for Improving Access to Health and Hygiene for People with Disabilities: Two Case Studies from Nepal and Malawi

**DOI:** 10.3390/ijerph15122746

**Published:** 2018-12-05

**Authors:** Jane Wilbur, Tess Bright, Thérèse Mahon, Shaffa Hameed, Belen Torondel, Wakisa Mulwafu, Hannah Kuper, Sarah Polack

**Affiliations:** 1 International Centre for Evidence in Disability, London School of Hygiene & Tropical Medicine, London WC1E 7HT, UK; tess.bright@lshtm.ac.uk (T.B.); Shaffa.Hameed@lshtm.ac.uk (S.H.); Hannah.Kuper@lshtm.ac.uk (H.K.); Sarah.Polack@lshtm.ac.uk (S.P.); 2 WaterAid, London SE11 5JD, UK; ThereseMahon@wateraid.org; 3 Environmental Health Group, Department of Clinical Research, London School of Hygiene & Tropical Medicine, London, WC1E 7HT, UK; Belen.Torondel@lshtm.ac.uk; 4 Department of Surgery, College of Medicine, University of Malawi, Blantyre, Malawi; wakisamulwafu@gmail.com

**Keywords:** people with disabilities, access to health care, developing countries, menstrual hygiene management, hearing loss, hearing impairment, intellectual impairment, carers

## Abstract

Limited evidence exists about how to design interventions to improve access to health care for people with disabilities in low and middle-income countries (LMICs). This paper documents the development of two behaviour change interventions. Case study one outlines the design of an intervention to improve uptake of referral for ear and hearing services for children in Malawi. Case study two describes the design of an intervention to improve menstrual hygiene management for people with intellectual impairments in Nepal. Both followed existing approaches—Medical Research Council Guidance for developing and evaluating complex interventions and Behaviour Centred Design. The purpose is to demonstrate how these frameworks can be applied, to document the interventions developed, and encourage further initiatives to advance health services targeting people with disabilities. Important components of the intervention design process were: (1) systematic reviews and formative research ensure that interventions designed are relevant to current discourse, practice and context; (2) people with disabilities and their family/carers must be at the heart of the process; (3) applying the theory of change approach and testing it helps understand links between inputs and required behaviour change, as well as ensuring that the interventions are relevant to local contexts; (4) involving creative experts may lead to the development of more engaging and appealing interventions. Further evidence is needed on the effectiveness of these types of interventions for people with disabilities to ensure that no one is left behind.

## 1. Introduction

One billion people are estimated to have a disability worldwide, and more than 80% live in low and middle income countries (LMICs) [1]. People with disabilities are considered to be those who have “long-term physical, mental, intellectual or sensory impairments which in interaction with various barriers may hinder their full and effective participation in society on an equal basis with others” [2]. Evidence suggests that disability is linked to poverty in a cycle, whereby poverty increases the risk of disability, and disability increases the risk of poverty [3,4]. Through the underlying impairment, poverty and other mechanisms, people with disabilities are likely to experience poorer health than people without disabilities [1]. People with disabilities have the same healthcare needs as those without disabilities (e.g., access to vaccinations, routine health checks, HIV services, water, sanitation and hygiene, sexual and reproductive health). In addition, people with disabilities may require access to specific specialist services such as rehabilitation and assistive devices [5]. Overall, therefore, the need for healthcare services may be higher among people with disabilities, but some evidence suggests that their access to these services is poorer than for people without disabilities [1]. This may be due to the barriers faced in accessing health services, such as stigma, lack of accessible transportation, or lack of trained health professionals. Therefore, overcoming these challenges, and improving access to health and health-related services is of vital importance. 

Improved access to health services has the potential to maximise functioning, improve participation in a range of activities, and improve quality of life for people with disabilities [6]. Equitable access to health services for people with disabilities is essential to achieving Universal Health Coverage and for fulfilling the United Nations Convention for the Rights of People with Disabilities (articles 25 and 26) [5]. Further, the Sustainable Development Goals adopted in 2015, clearly state that no-one shall be left behind in the global push to attain these goals [7]. Despite this, there are very few examples in existing literature on the steps taken to design interventions to improve access to health and health-related services for people with disabilities in LMICs. Previous reviews on interventions to improve access to health services for children in LMICs have found no studies focusing on children with disabilities [8,9]. There is evidence from a wide range of LMICs suggesting that people with disabilities face substantial barriers to accessing care, which are often complex and interacting, arising across all dimensions of access – from the individual-level to health system and policy levels [10,11,12,13,14]. Addressing these multi-dimensional barriers, needs a multifaceted or ‘complex’ intervention approach [15]. A complex intervention can be one which: targets multiple groups or organisational levels; has a number of interacting components; or addresses a number of behaviours [15]. This paper describes the development of two behaviour change interventions designed to improve access to health or health-related services in Malawi and Nepal drawing on the Medical Research Council’s framework for designing complex interventions and the Behaviour Centred Design model to develop interventions. The purpose is to demonstrate how these frameworks can be applied, to document the interventions developed and to encourage further initiatives to advance health services targeting people with disabilities.

## 2. Approaches to Designing New Interventions 

Several approaches exist for designing complex interventions to address public health issues. The case studies described in this paper draw on two key approaches in their development: The Medical Research Council (MRC) framework for designing complex interventions and Behaviour Centred Design (BCD) [16,17]. These two approaches are complementary. This section provides a brief summary of each. For a more comprehensive overview, see the MRC and BCD guidance [16,17]. 

The MRC framework suggests that first a systematic review should be carried out to understand what does or does not work (identifying the evidence base). Then an appropriate theory for the intervention should be developed drawing on the existing evidence, and also supplementing this with additional research, for instance qualitative research with the target population to understand need (e.g., people with disabilities) [17]. Next, participatory workshops with key stakeholders are recommended to develop a Theory of Change (ToC) and potential solutions, which are then developed, pilot tested for feasibility and acceptability, and studied in a wider trial to understand impact. The ToC is a pragmatic framework which describes how an intervention is expected to influence change. It is usually developed in collaboration with key stakeholders and includes a series of hypothesised causes and effects which lead to the intended impact. Indicators of success are developed for each stage to measure progress.

BCD follows a similar stepwise process, including analysing available literature on the topic, conducting formative research and developing a ToC, but it includes a greater focus on understanding the underlying drivers of behaviour [16]. Formative research methods proposed by the BCD focus on observation rather than asking participants to describe their behaviour through in-depth interviews or focus group discussions [18]. This is because many people are not consciously aware of what drives their behaviours [16,18]. 

These approaches have been used for designing and evaluating interventions targeting a range of health related behaviours, including hand washing with soap, food hygiene and nutrition, however there is little evidence of how this works for people with disabilities [19,20,21,22]. People with disabilities may face unique barriers to accessing health services, which presents additional challenges to designing interventions to suit their needs. For instance, people with disability may face discrimination from providers, or communication barriers. They may also incur greater financial barriers to accessing health care as a result of additional health care needs (e.g., rehabilitation or specialist health care) [23]. 

### 2.1. The Medical Research Council’s Framework

The MRC definition of a complex intervention includes interventions that have many interacting components, or those that target a number of behaviours that are difficult to change, and those that result in a number of possible outcomes. The MRC’s guidance for developing complex interventions include the following steps [15,17]:

1. Identifying the desired outcome
2. Identifying how to bring about change based on theory and evidence
3. Testing the feasibility of the intervention to ensure that it is acceptable and can be delivered as intended
4. Evaluation of the intervention through both impact and process evaluations

### 2.2. Behaviour Centred Design

**Figure 1:**
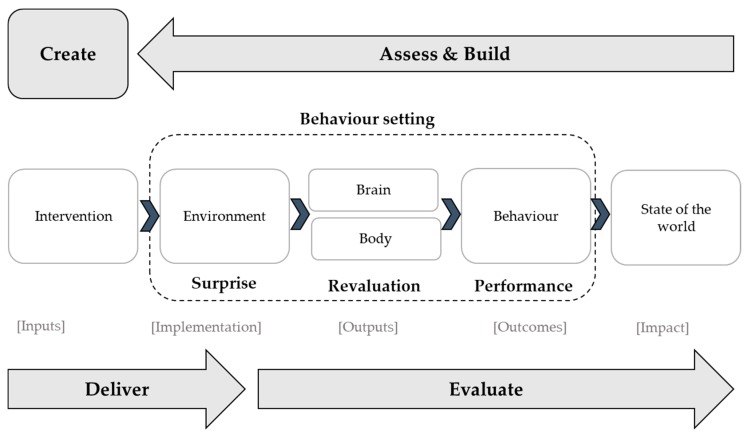
Behaviour Centred Design (BCD) approach (adapted from Aunger et al. 2016) [16].

BCD was developed by the London School of Hygiene and Tropical Medicine (LSHTM) and is grounded in behaviour science and design theories [16]. BCD recognises that interventions cannot directly affect behaviour. Instead they work through a series of causal links from their implementation in the environment through the bodies and into the brains of the target individuals to influence their behaviour and meet the desired impact, such as improved health. It focuses on human motives (e.g., affiliation: desire to be part of a group or status: desire to improve one’s social standing), which are used to increase the reward associated with the target behaviour. The series of causes and effects as a result of the intervention is captured in a ToC (Figure 1) [16].

The process of developing a behaviour change intervention is shown through the arrows around the outside: Assess, Build, Create, Deliver and Evaluate. The purpose of Assess is to understand what is already known about the target behaviour. Build includes formative research to enhance context specific knowledge about the target behaviour in relation to the target population. Create involves a team of stakeholders developing the behaviour change intervention, including those involved in the formative research, as well as practitioners and creative/marketing experts to ensure products developed are relevant, appealing and user-friendly. The intervention is then delivered and evaluated. The process through the center of Fig 1. shows the ToC, starting with the intervention and ending at the “state of the world”—the desired impact. 

Table 1 provides a comparison of the steps used in intervention development in the BCD and MRC frameworks. Although both frameworks were drawn upon in each of the case studies, the findings are presented using the BCD steps for simplicity. This paper focuses on the intervention development (ABC) part of the process; deliver (D) and evaluation (E) components will be addressed separately. 

The approaches used in each case study differed somewhat in the approach taken to create the interventions. In Nepal, the BCD was followed more closely because it was designed by environmental health researchers for developing complex water, sanitation and hygiene behaviour change interventions, and it has been applied in hygiene promotion programmes in Nepal and other south Asian countries with some success [21,22]. In Malawi, the intervention development was informed by the well-recognised MRC guidance. For the intervention design process the MRC is less prescriptive, so in addition to the formative stages of research, other frameworks were drawn from in the creative stage, including the BCD manual and the Behaviour Change Wheel [24].

## 3. Case Studies 

### 3.1. Case Study 1: Designing an Intervention to Improve Uptake of Referral for Ear and Hearing Services for Children in Malawi 

Ethical approval for this research was obtained from LSHTM (code 14433), and the College of Medicine Research Ethics Committee (COMREC) in Malawi (code P.09/17/2278). Informed consent in the local language was obtained for all components of the study involving research participants.

#### 3.1.1. Study Aim and Setting

This study aimed to develop an intervention to improve uptake of referral for children with ear and hearing conditions in Thyolo district, Malawi. Previous evidence from other LMICs had shown poor uptake of referral for children with disabilities [10,11]. Low uptake of ear and hearing services had also been observed anecdotally by practitioners within Malawi. The target population were children needing a referral, either on account of: disabling hearing loss (average of moderate or worse hearing in the better hearing ear according to the World Health Organisation [25]), or ear diseases which have the potential to lead to permanent disabling hearing loss if untreated (i.e., conditions that cannot be treated at health centres such as chronic suppurative otitis media, and dry perforations). Thyolo district is a poor rural district of Southern Malawi with a population of approximately 500,000. 

#### 3.1.2. Assess: Systematic Review

A systematic review was conducted on improving access to health services for children in LMICs, in order to understand what works elsewhere. The results of this review have been published previously [8,9]. In brief, this review considered evidence for effectiveness of the following interventions to improve healthcare uptake: delivery of services close to home, service level improvements, educational programmes, text message reminders, and provision of incentives. This review concluded that text message reminders and delivery of services close to home showed the most promising results. The findings for educational interventions were mixed, with some, but not all finding positive results. The review concluded further evidence was needed, however it provided vital background to what has been attempted previously in other LMICs.

#### 3.1.3. Build: Formative Research

An initial study using the Key Informant Method in Thyolo district was carried out in 2015, whereby community health workers (known in Malawi as Health Surveillance Assistants) identified children with hearing impairment and/or ear disease [26,27]. Screening camps were then carried out in the district during which children were examined by ear and hearing professionals. For children who needed surgery, hearing aids or further assessments, referrals were made to Queen Elizabeth Central Hospital (QECH) in Blantyre. QECH is the closest health service to Thyolo that provides specialist ear and hearing services. Evidence from a follow up survey found that uptake of referrals for those in need was extremely low (<5%). We conducted a mixed methods study to explore reasons for low uptake of referrals. This involved structured questionnaires administered to 170 children who were referred to QECH, followed by in-depth interviews with a sub sample of 23 caregivers of children who did not attend QECH following referral, and 15 stakeholders involved in ear and hearing care in Malawi. This research was conducted between June–August 2016 and the results have been published previously [12]. The key barriers reported included:

1. Fear and uncertainty about the referral hospital
2. Procedural problems within the camps leading to lack of understanding about the referral
3. Distance to the hospital
4. Low awareness and understanding of hearing loss
5. Lack of and cost of transport

#### 3.1.4. Create: Designing the Intervention

##### (1) Focus Group Discussion and Theory of Change Development

A focus group discussion was held with five caregivers from the original Key Informant Method study. The focus group was facilitated by a local researcher who was guided by an experienced qualitative researcher. The purposes of the focus group were to feed back the findings from the formative research and discuss strategies (interventions) to address the key barriers. This focus group formed an important part of the create step, as we wanted to ensure the caregivers could share their views in a comfortable environment, amongst peers facing similar care-seeking challenges. In order to understand more detail about decision making about care seeking and inform the intervention design, discussion also covered this topic. The caregivers validated findings from the formative research, and raised other key issues. Caregivers suggested that hearing impairment is not a priority for many families as there are no immediate complications like there are with acute conditions such as malaria. Regarding decision-making, the caregivers asserted that decisions about seeking care are made at home in a family meeting. Caregivers discussed a range of interventions that could address the barriers which were incorporated in to the next step (participatory workshop).

Following the focus group, a participatory workshop was held to present research findings from the formative research, focus group discussion, and systematic review and to develop ideas on how to overcome the barriers identified and improve uptake. To do this, a ToC workshop was conducted at QECH in Blantyre. In total, 19 stakeholders working in, or with an interest in ear and hearing care in Malawi attended the workshop. This included ear and hearing health professionals and administrative staff/project coordinators from QECH, Health Surveillance Assistants from Thyolo, a Ministry of Health official, a representative from Malawi Counsel for the Handicapped, a representative from Malawi National Association for the Deaf, other Disabled Persons Organisations (DPOs), and caregivers whose children were referred in the 2015 Key Informant Method study. For the workshop, participants were divided in to three groups, ensuring a balance between different sectors, whilst also ensuring that group members could all communicate with each other (some participants did not speak English/Chichewa). Three experienced facilitators ran the workshop (TB, RT, WM), two of whom were Malawian. First the research on uptake and barriers to referral services was presented, and additional findings from the focus group discussion with carers incorporated. Following this, we used a traditional ToC development approach [28], which included the following steps:

- Developing a long-term goal for the project
- Backwards mapping from the long-term goal to outcomes and intermediate outcomes required to reach the long-term goal
- Discussing possible activities (interventions) to achieve the prioritised outcomes
- Prioritisation of suggested activities (interventions) in terms of cost, feasibility, acceptability and sustainability

The long-term goal for this project—and the target behaviour to change - was improved uptake of ear and hearing referrals for children. As attending QECH is often not a one-off event, with most people needing additional appointments beyond the initial referral, our ultimate goal was that attendance at follow-up appointments was also sustained, resulting in improved ear and hearing health. To develop outcomes, each of the barriers identified in the formative research were reversed e.g., “lack of transport” became “transport is available”. Thus, five key outcomes related to the barriers identified in the research were developed.

##### (2) Proposed Interventions 

Table 2 shows the five outcomes (addressing each barrier) and the interventions that were proposed to achieve these. Members of the focus group and ToC workshop particularly emphasised that learning from peers who had been through the experience of attending QECH would be encouraging for caregivers. 

A prioritisation task was held whereby each member of the group voted on their top three interventions—with consideration to costs, feasibility, acceptability, and sustainability. Through this task, a consensus was reached to focus on interventions 1–3 in Table 2 (i.e., those that address fear about the hospital, awareness and understanding about ear and hearing health, and information about the referral). Provision of transportation was not considered to be a sustainable option by all members of the ToC workshop, including caregivers, due to the high perceived long-term costs, despite transportation being a key barrier. 

Building on the recommendations from the workshop, consultation with experts in educational interventions, and the evidence obtained from the systematic review, the following multi-component intervention was agreed:

1. A photograph/pictorial information booklet providing information about the process of going to the hospital for ear and hearing services
2. Counsellors trained to deliver information booklet in camp settings, including one “expert” mother (i.e., mother of a child with ear and/or hearing issue who has attended QECH for referral previously) who would provide peer support and a community health worker
3. Text message reminders for caregivers who had been referred to QECH

Each component of the intervention aimed to address the barriers raised in the formative stages of the research, and drew on the results of the systematic review, and existing behaviour change techniques [24]. The final ToC can be found in supplementary material (Figure S1). The rationale behind each component of the intervention can be found in Supplementary Material (Table S1).

##### (3) Engagement with Creative Team

A London-based creative agency, RE-UP (https://thisisreup.com/), was contracted to create the information booklet. The agency was briefed on the background of the project, the Malawi context, and the intervention proposed. The key sections of the booklet that the agency were tasked with developing included: a story of a child going to QECH with their caregiver, directions to the hospital and department, and a plan of action for counselors to discuss with each caregiver.

The development of the booklet involved an iterative process. Firstly, to tell the story of a family attending QECH, interviews were conducted with two caregivers of children from Thyolo who had previously attended QECH—one for their child’s ear surgery and one for hearing aid fitting. These caregivers were selected from QECH registries. Details were gathered about their child, the referral process, the journey and their experiences at the hospital. In addition, photographs were taken by a local photographer of the caregivers, a typical village in Thyolo, the hospital, ear and hearing health professionals, and landmarks that would be important for caregivers to recognise on their journey. These stories and photographs were used to draft the first version of the booklet. The creative agency suggested that a storyline would work well with illustrations of the child and caregiver attending QECH, rather than photographs, so that the intervention was more engaging and surprising. An element of surprise is recommended in the BCD approach to ensure people will pay attention to it and assist engagement in the behaviour change process [16]. 

An iterative process of consultation and adaptation of the booklet was undertaken, with recommended changes incorporated by RE-UP between each of the following consultation stages:

- Draft 1 and 2 reviewed by LSHTM researchers
- Draft 3 reviewed by eight stakeholders from Malawi (six from original ToC workshop)
- Draft 4 reviewed by target population (caregivers of children with ear and hearing issues from Thyolo district) through a focus group discussion). Caregivers were asked to reflect on suitability of the images, comprehensibility of the text, and usefulness of the components of the booklet.

##### (4) Intervention 

**Figure 2:**
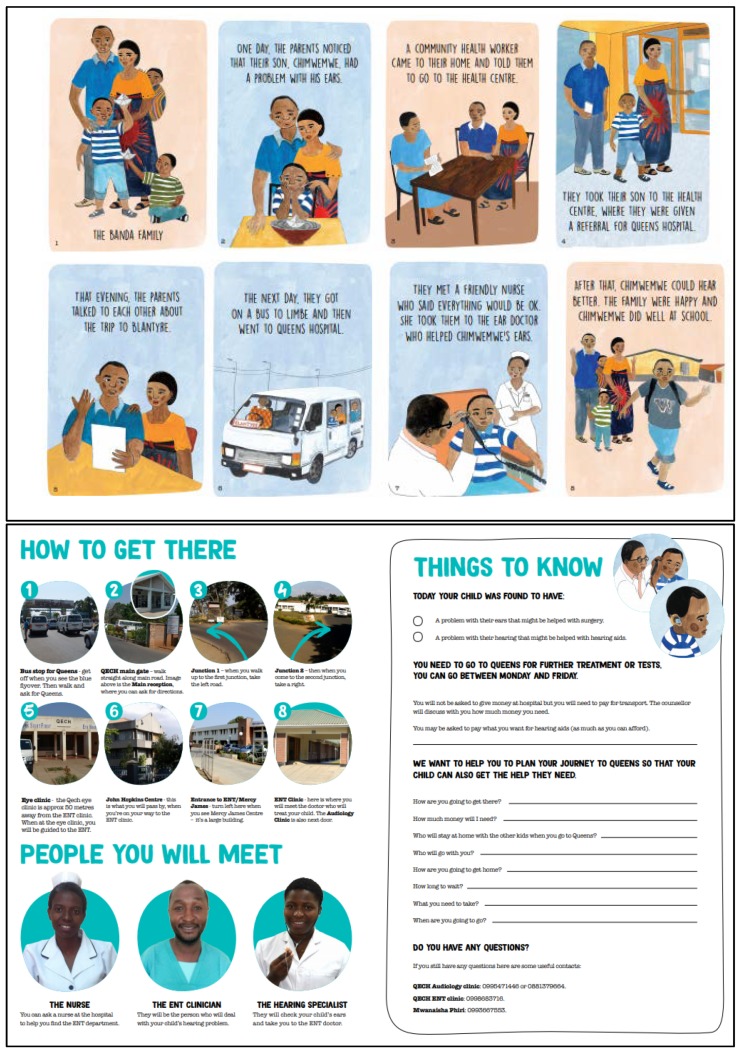
Final English version of the information booklet for Malawi intervention, before translation into Chichewa. The set of illustrated images at the top show the storyline, and the bottom shows further information about how to get to the hospital, the people that will be met, and a section on action planning (things to know) which was tailored to the individual family. The booklet was folded down to A6 format with page 1 of the booklet showing the first panel of the story (The Banda Family).

**Figure 3:**
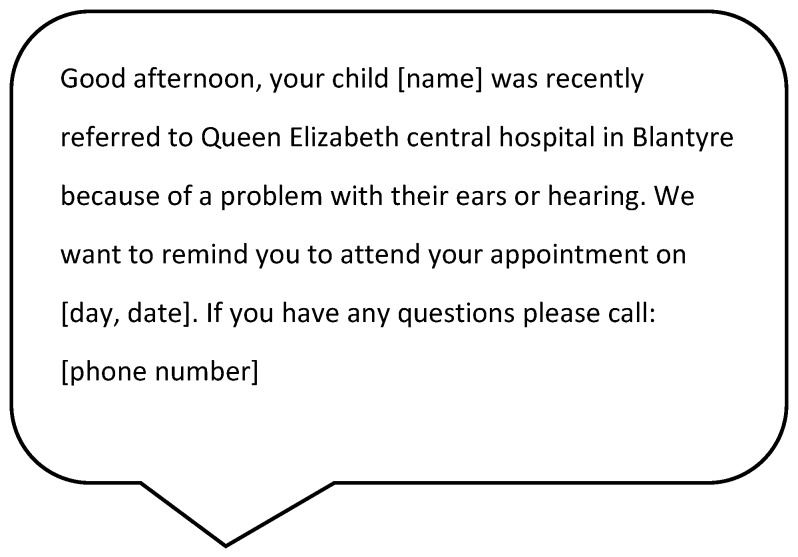
Text message reminder (English version).

The final components of the intervention include:

- A booklet with three main parts (Figure 2): (1) An illustrated storyline of “The Banda Family” going through the process of being referred and attending the referral at QECH; (2) Information on how to get to the hospital including photographs of key landmarks that caregivers would see on the way to the ENT department; (3) Action planning stage that was tailored to each caregiver—including how they plan to go, how much money they need, and what they need to take with them. This booklet would be delivered by a trained “expert mother” (i.e., mother of a child with ear and/or hearing issue who has attended QECH for referral previously) at the point at which the referral was made (in camps).
- A text-message reminder to be sent two weeks after the referral (Figure 3).

#### 3.1.5. Deliver: Feasibility Studies 

In the next stage of the development process, the feasibility of the intervention will be assessed in a study with 30 members of the target population. Mixed methods approaches will be used. Research will seek to understand from stakeholders whether the intervention is appropriate, and what adjustments can be made. Following this, the intervention will be adapted, and a trial setting to understand impact.

### 3.2. Case Study 2: Designing an Intervention to Improve Menstrual Hygiene Management of People with Intellectual Impairments in Nepal

Ethical approval was granted from the Nepal Health Research Council (code 102-2017 and 39-2018 and the LSHTM Ethics Board (code 12091 and 15703). 

#### 3.2.1. Study Aim and Setting 

This study aimed to improve menstrual hygiene management (MHM) by adolescents and young people with disabilities and their carers in Kavrepalanchok district, Nepal. MHM is defined as ‘women and adolescent girls using a clean menstrual management material to absorb or collect blood that can be changed in privacy as often as necessary for the duration of the menstruation period, using soap and water for washing the body as required, and having access to facilities to dispose of used menstrual management materials. They understand the basic facts linked to the menstrual cycle and how to manage it with dignity and without discomfort or fear’ [29]. MHM also involves addressing harmful social beliefs and taboos surrounding the issue. There are concerns that people with disabilities face particular difficulties in MHM, yet few interventions are available to address this issue. This was a collaborative study between the LSHTM and WaterAid, with funding from the Bill and Melinda Gates Foundation. 

#### 3.2.2. Assess: Systematic Review

Initially a systematic review of peer-reviewed literature was conducted to understand the MHM requirements of people with disabilities in different settings. The results of this review will be reported separately. In brief, few eligible studies were identified, but in general the review showed that people with intellectual impairments faced particular difficulties in MHM, yet few interventions are available to support MHM in this group. 

#### 3.2.3. Build: Formative Research

Formative qualitative research was conducted in the Kavrepalanchok district, to understand the specific MHM requirements of (1) adolescents and young people with a disability and the barriers they face in managing their menstruation hygienically and with dignity; and (2) carers who support these people during menstruation. 

Twenty women and girls with disabilities aged 15–24 who menstruated, were identified using the Washington Group Short Set of questions [30]. Twelve carers who support these people during their menstrual cycle and 13 policy makers and practitioners focusing on water, sanitation, hygiene, disability and/or sexual and reproductive health at the district or national levels also formed the sample. 

Observing all menstrual hygiene management related behaviours is difficult to do as they are private, so a range of participatory methods were applied: in-depth interviews, PhotoVoice, market survey and product attribute assessments of the menstrual products, and accessibility and safety audits of the menstrual hygiene management facilities. In-depth interviews were conducted instead of focus-group discussions, so that individual’s nuanced experiences could be explored in detail and with a level of sensitivity that group interviews are less suited to. 

Findings from the formative research will be reported elsewhere, however they supported the results from the systematic review that people with intellectual impairments faced the greatest difficulties in MHM. Whilst people with physical impairments faced barriers to MHM many of these were similar to the barriers experienced by this group when accessing water and sanitation facilities, on which considerable research has been published [31,32,33]. Additionally, the few initiatives to develop MHM interventions for people with disabilities in LMICs focused on people with visual and hearing impairments [34,35]. These findings formed the rationale for the focus on people with intellectual impairments for the intervention. The key barriers to MHM among people with intellectual impairments that emerged from the formative research were:

- Limited MHM training, information and support. MHM information is often withheld from people with an intellectual impairment because of perceived levels of understanding. Carers have no support, information or guidance on how to manage another person’s menstrual cycle, leading many to feel overwhelmed.
- Limited ability to communicate verbally, understand menstruation and related social norms. Some people with intellectual impairments are unable to communicate verbally that they have pre-menstrual symptoms and may not understand the reason for these. Carers do not always provide pain relief for menstrual cramps. During menstruation some participants are frightened, withdrawn, or refused to eat. Some showed their menstrual blood or hygiene products to others and are abused for doing so.
- Poor access to existing MHM interventions. In Nepal, MHM interventions are predominantly delivered through school and community mechanisms. Many research participants with an intellectual impairment do not attend school so cannot access these. Some carers are unable to leave their home because of caring duties, so are unable to access the MHM information delivered through the community.

#### 3.2.4. Create: Designing the Intervention

##### (1) Focus Group Discussion and Theory of Change Development

Drawing on the systematic review and formative research findings, a ToC was developed by the lead researcher (JW) using the BCD approach, instead of developing it collaboratively at a stakeholder meeting due to resource constraints. The theory considers the target groups and behaviours, human motives, and intervention activities. The final theory is found in the supplementary material (Figure S2). Strategies to improve MHM of people with intellectual impairments were explored through the systematic review, and a scoping review of MHM resources developed for people with disabilities for high-income country settings. 

JW shared the formative research findings with carers of people with intellectual impairments from the original sample at individual meetings. A problem tree was used to show the root causes of the issues and their effects. For instance, a lack of MHM information and support to people with intellectual impairments can lead to feelings of fear and confusion when they menstruate. Carers were asked semi-structured questions to understand if they agree with these findings, which they all did. The research findings were then disseminated to key stakeholders at a meeting in Kathmandu. 

##### (2) Engagement with Creative Team 

A creative team was set up to develop the MHM behaviour change intervention for people with intellectual impairments in Nepal. The team included a local artist and a marketing professional, as well as people experienced in developing interventions in Nepal (WaterAid, Mitra Samaj), working with people with disabilities (the Down Syndrome Society Nepal), and implementing MHM programmes in the Kavrepalanchok district (KIRDAC, CIUD and government social mobilisers). The creative team attended the dissemination of the formative research findings meeting, and then developed the intervention over 3.5 days during a participatory workshop. 

JW and TM led the workshop, which followed the intervention development stages set out in the BCD manual [22]. In summary, the formative qualitative research findings were clustered into six overarching themes: (1) Independence: MHM information at the right time and place, (2) Comfort and confidence: providing pain relief, pads, understanding and communicating pain, (3) Reproductive rights: most girls menstruate, including girls with intellectual impairments, and everyone has a right to understand their bodies and make choices about them, (4) Harmony in the household: providing emotional support, (5) Be recognised as a good citizen: a good citizen adheres to positive social norms/behaviours about MHM, (6) Love your body, love yourself, love your period: address harmful social norms and menstrual restrictions. The themes were then mapped against the human motives identified in the ToC.

The creative team then came up with a statement that connected each theme with the relevant target behaviour. Positive visions (known as “insights”) of what adoption of target behavious could lead to were then developed (e.g. a person with an intellectual disability can feel more comfortable and confident to manage bleeding, pain and stress if they use effective menstrual products). An example of how the insight for the ‘comfort and confidence’ theme was developed is included in the supplementary materials (Table S2). The strongest insight was chosen, using criteria pre-defined in the BCD manual. This insight was developed into the intervention concept. 

##### (3) Intervention Concept

The intervention concept is a narrative which brings the intervention components together under a single package. The aim was to create a concept that captured the audience interest, evoke an emotional response and encourage the adoption of the target behaviours. The intervention concept developed was: 

Bishesta (meaning ‘extraordinary’ in Nepali) is a young woman with an intellectual impairment with hidden, and extraordinary talents. She gets her menstrual product and pain relief from her carers; she uses them properly. She feels comfortable and confident at home and in public. She lives a dignified life. She is loved by all her family members and everyone in the village. She is an inspiration to others as she is learning to be as independent as possible. Whenever she needs support to understand the changes she faces when growing up, Perana (meaning ‘motivation’ in Nepali) motivates and helps her. 

Perana provides information and emotional support to Bishesta. She also offers enough menstrual products and provides pain relief when Bishesta needs it. When Perana does this, Bishesta shows her love. Perana feels confident that Bishesta is more able to take care of herself. The family is happy and they are respected in the community.

**Figure 4:**
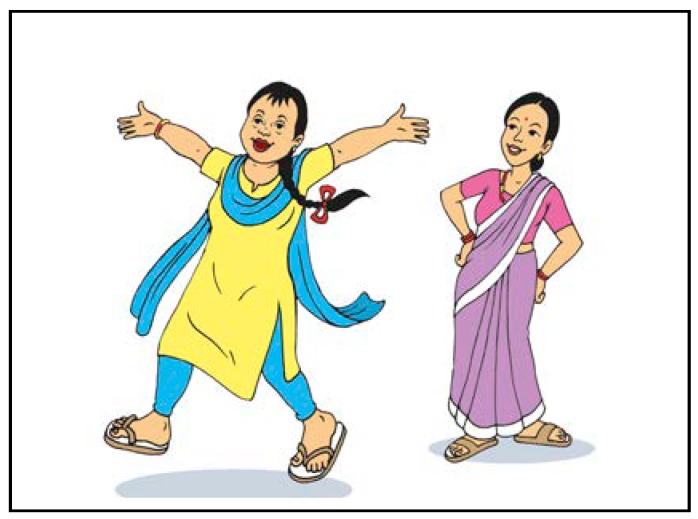
Bishesta and Perana.

Figure 4 shows the faces of the intervention: Bishesta and Perana.

The Down Syndrome Society Nepal (DSSN) advised on how to depict Bishesta, who has Down syndrome. Involvement of this DPO in the create stage was integral to developing a credible intervention, especially as the representative from the DSSN was a carer of a person with Down syndrome. The intervention is also branded for greater legitimacy; for instance, the brand colours are blue and yellow and its logo is on all the components. 

##### (4) Touchpoints

‘Touchpoints’ are the ways in which the target audiences should come into contact with the intervention. [18] All possible touchpoints were identified by the creative team. These were then discussed and ranked according to which were the most appropriate for the context and available resources. The chosen touchpoints for the Bishesta intervention were household visits and group training sessions delivered by staff members from the DSSN and Centre for Integrated Urban Development (CIUD). 

##### (5) Intervention

The intervention includes a menstrual hygiene pack for the person with an intellectual impairment containing: a menstrual storage bag (for inside home), menstrual shoulder bag (for outside home), a menstrual bin, a pain bangle and visual stories. Carers receive a menstrual calendar. Each participant also receives a branded key ring, badge and mirror. Table 3 shows the target group for each intervention component, the target human motive, and the required training for each component. Each component and training activity encourages the adoption of the target behaviours. Further details about each of the intervention components are described in the following section.

a. Intervention component details

The menstrual storage bag contains reusable menstrual pads. It is for use inside the home and ensures the participant can always access a menstrual product. The menstrual shoulder bag is designed for use outside the home. It includes reusable menstrual pads and a small waterproof zip bag for a soiled menstrual product. 

The menstrual bin is plastic with a swing lid, so can be wiped clean. It should be placed near the participant’s bed for disposing of used menstrual products. 

The pain symbol bangle has a red, orange and yellow coloured strip. These represent severe, moderate and mild pain respectively. These allow people who are unable to communicate verbally to request pain relief from carers by pointing to the relevant colour.

Two visual stories: (1) ‘I change my pad’ is about Bishesta menstruating for the first time and how Perana supports her through this, (2) ‘I manage’ shows Perana helping Bishesta to understand she must not show her menstrual blood in public. The stories include all the target behaviours, human motives and bring the intervention concept to life (These visual stories are based on a method developed by Beyond Words (www.booksbeyondwords.co.uk)). 

Carers will be given a menstrual calendar to help track the menstrual cycle, including any changes in related behaviour of the person they support. Prompts for carers’ target behaviours are included at the bottom of the calendar. 

b. Training

The implementation of the intervention is planned as follows: implementers will deliver three MHM training modules over three months to groups of ten people with intellectual impairments and their carers. During the group sessions, peer-to-peer support for carers will be facilitated and menstrual hygiene packs will be distributed. 

JW developed monitoring indicators to track participant’s progress against adopting the target behaviours and using the menstrual hygiene packs. Implementers will visit participant’s homes to monitor progress against these indicators and offer ad-hoc support. Households that adopt the target behaviours will be recognised as ‘Bishesta households’ at the next group training session. 

The indicators are designed to be achievable to ensure that all participants can achieve this status. Implementers will also monitor attendance and the fidelity of the intervention delivery.

**Figure 5:**
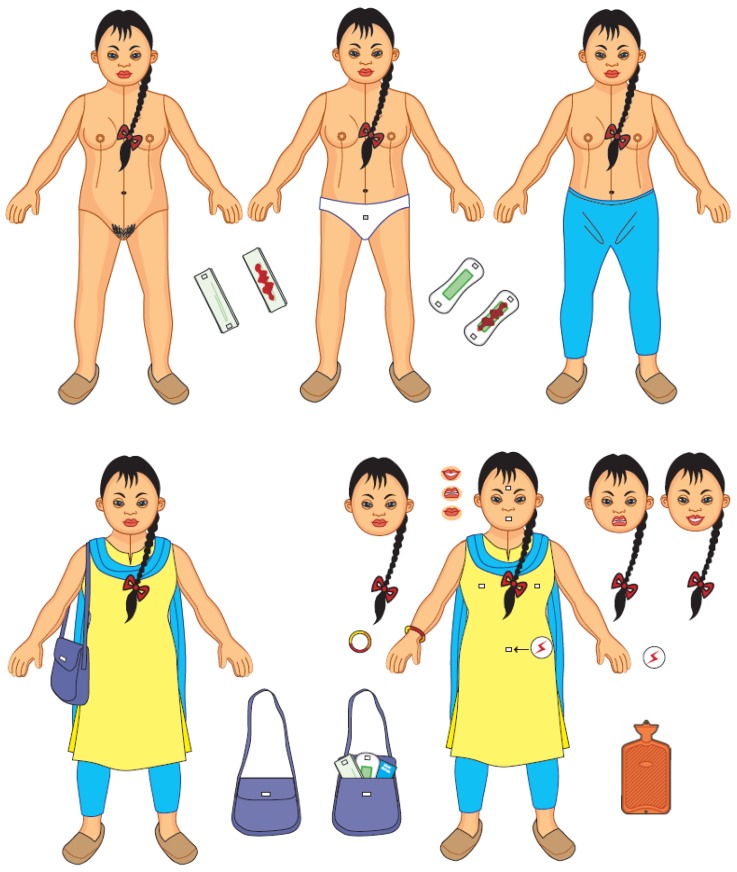
Large Bishesta doll.

A large Bishesta doll, which has a number of features to facilitate communication and develop skills in MHM, will be used in the training sessions (Figure 5). The doll has all the components included in the menstrual hygiene packs, so all participants can understand their purpose and practice using them with the doll before taking their packs home. In addition, the doll includes a range of other components, such as removable pain symbols that can be placed on the doll to show where discomfort may be felt, and a ‘clean’ and ‘soiled’ menstrual pad that participants can practice changing.

#### 3.2.5. Deliver: Feasibility Study

In the next steps, a pilot study will be undertaken to test the intervention components and training manual with implementers, four people with intellectual impairments and their carers. Materials will be revised based on participant’s feedback before implementation. A process evaluation and feasibility study will be conducted to assess the fidelity of the intervention, and feasibility and acceptability of the intervention. The intervention will then be adapted and/or delivered at a wider scale with an impact evaluation conducted at the end.

#### 3.2.6. Comparison of Intervention Design Processes 

The two case studies presented above used different approaches within the ‘build’ and ‘create’ stages. The projects were distinct, each with their own set of resource constraints, epistemological positions, researchers and collaborating partners who influenced the research design. In Nepal, one priority was to develop a multi-component intervention that systematically disrupted the environment, body and brain of the target group. Another was that a local creative team actively participated in the whole ‘create’ stage, which led to a strong level of ownership. In Malawi, the priority was to create a low-cost multi-component intervention within a short time frame which could be easily incorporated in to existing programmes. Unlike Nepal, the Malawi project used London-based creative agency was employed due to substantial time constraints, leading to inability to identify appropriate local designers within the specific time-frame. 

The nature of the behaviours explored were vastly different in each setting. This determined the data collection methods applied in the ‘build’ and ’create’ stage of the research. For instance, in Nepal menstrual hygiene management behaviours are extremely private, so a number of participatory methods were applied to ensure participants could express themselves in different ways (e.g., PhotoVoice). In Malawi, semi-structured interviews and focus group discussions were used because the researchers felt that the topic could be spoken about openly. Table 4 provides a comparison of the processes followed in the two case studies.

## 4. Discussion

This paper describes the process of designing multi-component behaviour change interventions for people with disabilities in LMICs using two unique case studies. Although the settings and purposes in the two case studies are vastly different, the steps used in each were broadly similar, drawing on the BCD and MRC approaches, including systematic review, formative research, participatory development of intervention and (in the future) assessment for feasibility and impact.

### 4.1. Key Lessons, Implications for Research and Practice

The BCD and MRC frameworks offer systematic approaches to developing interventions. In both case studies, the steps to develop an intervention outlined in the BCD manual for practitioners were found to offer clear and concise guidance [18]. Using this process to design interventions for people with disabilities highlighted some important aspects that could be applied in other settings and sectors, beyond public health. 

Firstly, conducting a systematic review of relevant literature meant the studies were relevant to current discourse. It also ensured learning from existing interventions so that work was not duplicated and successes could be built upon. For example, studies evaluating text message reminders generally found a positive effect on access to health services in LMICs for the Malawi case study. The formative research was valuable as it provided more in-depth contextual information to inform the intervention design. 

Secondly, both case studies sought to ensure people with disabilities, their carers and DPOs are at the heart of the process in each stage of the development. This is in line with the principle of ‘nothing about us without us’: no disability related policy or intervention should be designed without the full and direct participation of people with disabilities. The process also maximises the likelihood that the intervention developed is relevant and acceptable to the target audiences. There are also challenges with meaningfully engaging people with disabilities, which are discussed below.

Thirdly, applying the theory of change approach and testing it, helps understand links between inputs and required behaviour change as well as ensuring that the interventions are relevant to local contexts. This supports findings from a paper by de Silva et al., which found that stakeholder engagement in intervention development is helpful for context specific solutions using the theory-driven ToC process [36]. This process also ensured stakeholders had a common understanding of the final goal from the outset. In the future, the ToC process will allow authors to identify if the critical activities were delivered as intended. If they have been, the logical linkages between inputs and subsequent behaviour change can be tested. If behaviour change is achieved, this will suggest a positive causal link between inputs and outcomes [36]. 

Further, creative experts were involved in the product design and marketing process in order to create demand and ensure appealing and user-friendly interventions to encourage the adoption of target behaviours. In the Nepal case study, the intervention was branded for greater legitimacy and recognition. In Malawi, the creative agency was UK-based due to time and resource constraints limiting opportunities to engage local designers. Thus, pilot testing the materials with the target population was essential to ensure cultural relevance. In both case studies, the involvement of DPOs throughout the process was key in ensuring that the materials are appropriate for the target group, and therefore may improve stakeholder buy-in. 

There is limited evidence on intervention design with or for people with disabilities in the existing literature. However, in the field of mental health the MRC approach has been followed to develop a range of interventions. For instance, in Ethiopia, Asher and colleagues describe the development of a community-based rehabilitation intervention for people with schizophrenia using the MRC approach. They assert that the ToC allowed articulation of assumptions and development of culturally appropriate ways to improve functioning for people with schizophrenia through community-based rehabilitation [37]. The BCD has been applied in water, sanitation and hygiene interventions [22,38]. For example, the ‘SuperAmma’ campaign in India, which focused on improving handwashing with soap at critical times (i.e., after using the latrine, and before preparing food and eating), followed the ABCDE principles to design and implement the intervention. Results from the clustered randomised control trial reported an increase in handwashing with soap in the intervention group from 1% at baseline to 29%, 12 months post intervention [22]. 

In this paper we focus on the processes followed and which aspects are important for designing interventions for people with disabilities. We are unable to say whether the interventions developed in this research are effective as it is outside the scope of these case studies. Given this constraint, further studies are needed that evaluate the effectiveness of these types of interventions for people with disabilities. This evidence is vital to ensure that no one is left behind in the Sustainable Development Goal era. 

### 4.2. Challenges 

A number of common challenges were faced in the case studies. Although in both studies, every effort was made to engage people with disabilities, this is not a straightforward process. For example, there was some concern that inviting the target groups from the formative research sample (persons with disabilities and their carers) to the create workshop may be tokenistic. In both settings, the key stakeholders in the create workshop were educated professionals from DPOs, government and non-governmental organisations. The caregivers involved in the formative research were from poor rural areas in both settings and were less educated. These differences would have made equal input into theoretical discussions challenging. In an attempt to mitigate this and ensure in-depth understanding of carers views inclusion of voices in the create process, focus group discussions and in-depth interviews were conducted prior to the workshops in both case studies. In Malawi, the focus group discussions were held in addition to a wider workshop in order to ensure that caregiver’s perspectives on how to address barriers that were particular to their lived experience were meaningfully incorporated in to the intervention development. In the workshops in Malawi, we ensured that the makeup of the discussion groups included people that the caregivers would be comfortable with, for example community health workers. In Nepal, the creative team included a carer of a person with Down syndrome from the DSSN, so her unique perspective helped shape the intervention design. The intervention will be also be tested with carers (from similar socio-economic groups as those in the formative research sample) prior to implementation to ensure their continued input into the creative process. 

Further efforts are needed to ensure that people with disabilities actively and meaningfully participate in the whole process of intervention development. In both settings, resource constraints limited the levels of participation that was possible. Alternative participatory approaches, such as Body Mapping and using Feeling Dice, should be investigated to ensure that children with disabilities and people with intellectual impairments can receive and communicate information non-verbally and therefore participate more meaningfully in the intervention design [39,40,41]. These methods will be applied during the feasibility study in Nepal. Wickenden et al. are working on developing these methods for children with disabilities, with a focus on removing barriers that prevent children from sharing their views [42]. They argue that advocating for participatory research with children with disabilities without making adaptations for their needs could lead to unethical or tokenistic practices. 

## 5. Conclusions

The two case studies demonstrate how a systematic approach to designing context specific behaviour centred interventions for people with disabilities in LMICs can be applied. This approach could potentially be used elsewhere. Meaningfully involving people with disabilities in the whole intervention design process is vital. Future studies are needed that evaluate the effectiveness of these types of interventions for people with disabilities to ensure that no one is left behind.

## Figures and Tables

**Table 1:** Comparison between steps in Medical Research Council (MRC) and Behaviour Centred Design (BCD) frameworks.

	BCD	MRC
Step 1	Assess and build	Identifying the desired outcome
Step 2	Create	Identifying how to bring about change based on theory and evidence
Step 3	Deliver	Testing the feasibility of the intervention to ensure that it is acceptable and can be delivered as intended;
Step 4	Evaluate	Evaluation of the intervention through both impact and process evaluations.

**Table 2:** Outcomes of theory of change workshop.

	Barrier	Outcome	Proposed Interventions
1	Fear and uncertainty about the referral hospital	Reduced fear about hospital	Peer support/counseling Information about hospital procedures communicated effectively during outreach
2	Procedural problems within the camps leading to lack of understanding about the referral	Sufficient information about referral	Information provided through: - Peer support/counseling - Village health committees - Videos about the referral process - Text message reminders
3	Low awareness and understanding of hearing loss/hearing loss is not prioritised	Improved awareness and understanding about ear and hearing health; hearing loss is prioritised	Ear/hearing day advocacy event Education of “gatekeepers” in the community (e.g., community leaders)
4	Distance to the hospital	Service available closer to the community	Expand outreach camps in the community
5	Lack and cost of transport	Transport is available	Group transport provided with community escort

**Table 3:** Intervention components and training activities.

Relevant Intervention Component	Target Group	Target Behaviour	Human Motive	Relevant Intervention Training Activity
Menstrual storage and shoulder bag, menstrual bin	Person with an intellectual impairment	Use a menstrual product	Comfort, dignity	Bishesta doll, role play
Pain symbol bangle		Use pain relief for menstrual cramps	Comfort, reward	
Menstrual shoulder bag, visual stories		Does not show menstrual blood in public	Affiliate, dignity	Bishesta doll, role play ‘Reading’ visual stories with carers
Menstrual storage and shoulder bag, menstrual bin	Carer	Provide enough menstrual products	Nurture, affiliate, reward	Emo-demos (surprising and motivating demonstrations and activities), peer-to-peer support, competition to become ‘Bishesta households’, guiding the person they care for through Bishesta doll role play and ‘reading’ visual stories, household monitoring visits/ad-hoc support
Menstrual calendar, visual stories		Provide pain relief for menstrual cramps	Nurture, reward	
Menstrual calendar, visual stories		Provide emotional support	Nurture, reward	

**Table 4:** Comparison of the processes followed in Malawi and Nepal.

Stage	Malawi	Nepal
Assess	Systematic review of relevant literature	Systematic review of relevant literature
Build	Formative research: Key informant method Structured questionnaires In-depth interviews with caregivers and stakeholders	Formative research: Research team of women with and without a disability In-depth interviews PhotoVoice Market survey and product attribute assessment of menstrual products Observation (accessibility and safety audits of the menstrual management facilities)
Create	Focus group discussions with carers from the key informant method study Participatory workshop to develop a Theory of Change and design the intervention Engagement with a creative agency Intervention: guidance booklet, counseling, text message reminder	Theory of change development Formation of a creative team Problem tree analysis and in-depth interviews with carers from the formative research sample Stakeholder workshop to disseminate the formative research findings Creative team workshop to design the intervention and its delivery mechanisms Intervention: menstrual hygiene packs and training for carers and people with intellectual impairment
Deliver	Feasibility study	Feasibility study

## Supplementary Materials

The following are available online at http://www.mdpi.com/1660-4601/15/12/2746/s1, Table S1: Rationale for each component of the intervention in Malawi, Table S2: Example: Insight development for the ‘comfort and confidence’ theme, Figure S1: Theory of change for improving uptake of referral for ear and hearing services in Malawi, Figure S2: Theory of Change for the menstrual hygiene behaviour change intervention in Nepal.
