# Supplementary material for: Developing Behaviour Change Interventions for Improving Access to Health and Hygiene for People with Disabilities: Two Case Studies from Nepal and Malawi
Source: Int J Environ Res Public Health. 2018 Dec 5;15(12):2746. doi: 10.3390/ijerph15122746 (PMC6313611; doi:10.3390/ijerph15122746)
Supplement: Supplementary file 1 [file ijerph-15-02746-s001.zip › New folder/Figure_S1.pdf]

Theory of change for improving uptake of referral for ear and hearing services

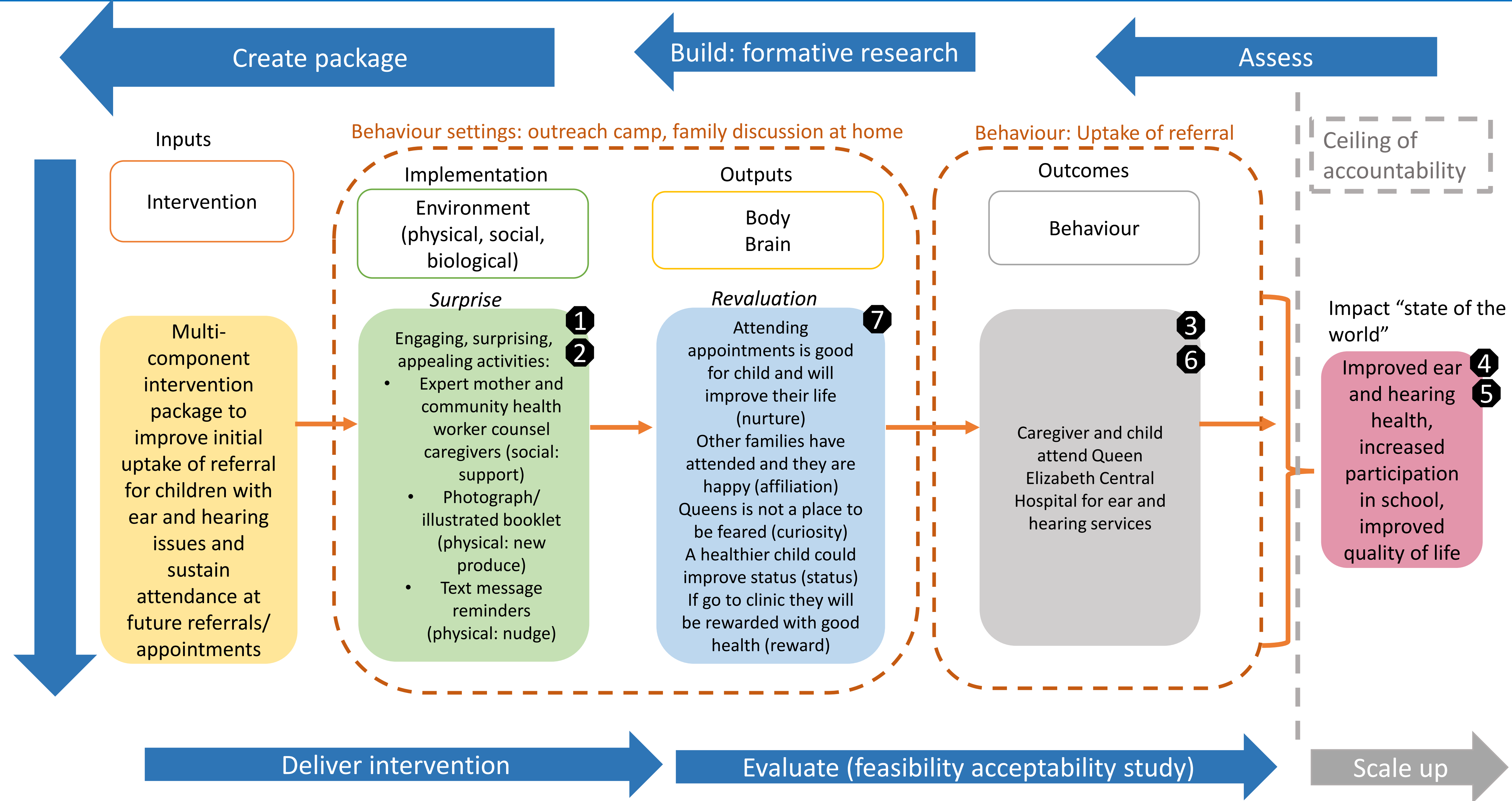

Assumptions

1 Adequate time allocated for counselling in camps available
2 Parents have mobile phone access
3 Caregivers are aware of transport services/services are available and have the funds to attend
4 ENT/audiologists have knowledge, skills and equipment to treat children attending the referral
5 Treatment and rehabilitation is effective
6 Parents have a good experience seeking care, and the doctors at Queens provide adequate counselling
7 Intervention successfully elicit desired responses
