# Supplementary material for: Developing Behaviour Change Interventions for Improving Access to Health and Hygiene for People with Disabilities: Two Case Studies from Nepal and Malawi
Source: Int J Environ Res Public Health. 2018 Dec 5;15(12):2746. doi: 10.3390/ijerph15122746 (PMC6313611; doi:10.3390/ijerph15122746)
Supplement: Supplementary file 1 [file ijerph-15-02746-s001.zip › New folder/Table S2.pdf]

Table S2: Example: Insight development for the 'comfort and confidence' theme.

Findings	Theme	Target behaviours	Statement connecting the theme with relevant behaviour/s	Insight	Motives
Persons with disabilities: want to feel more comfortable; may not understand the purpose of a menstrual product; may be sent home from school if unable to manage menstruation; may go out with blood stained clothes.<br><br>Carers: want to be a good carer; need to earn an income, so might leave the person they care for at home all day with a very thick menstrual cloth on; they have many tasks and may be too tired to change the menstrual product at the end of the day; might not always provide pain relief.<br><br>General: there is a belief that pain relief tablets can damage health, so many people do not take them; some people use natural remedies to manage menstrual cramps, such as a hot water bottle or tying a piece of cloth tightly around the abdomen.	Comfort and confidence: pads, pain relief, understanding and communicating pain.	Disabled person: uses a menstrual product when needed, uses pain relief; does not show menstrual blood in public.<br><br>Carer: provides enough menstrual products, provides pain relief and provides emotional support and love.	Managing menstrual blood and cramps can be made easier and more comfortable for disabled persons by using effective products and pain relief. Carers need to be able to provide these and give emotional support.	A good carer supports a person with an intellectual disability to be as independent as possible during menstruation. A person with an intellectual disability can feel more comfortable and confident to manage bleeding, pain and stress if they use effective menstrual products and pain relief and their carer provides emotional support, advice, pain relief and menstrual products.	Comfort, dignity, reward, affiliation
