# Supplementary material for: Developing Behaviour Change Interventions for Improving Access to Health and Hygiene for People with Disabilities: Two Case Studies from Nepal and Malawi
Source: Int J Environ Res Public Health. 2018 Dec 5;15(12):2746. doi: 10.3390/ijerph15122746 (PMC6313611; doi:10.3390/ijerph15122746)
Supplement: Supplementary file 1 [file ijerph-15-02746-s001.zip › New folder/Table_S1.pdf]

*Table S1: Rationale for each component of the intervention in Malawi*

| Intervention component | Behaviour change technique [1] | Hypothesised mechanism |
|---|---|---|
| Booklet | Instruction on how to perform behaviour (uptake of referral) | Providing directions and creating a tailored plan of action demonstrates to caregivers what needed to be done to attend, and familiarises them with the process to help overcome lack of understanding about the referral. |
| | Social comparison | The storyline shows other people going through the process of taking up referral and therefore caregivers compare themselves to the family in the booklet. This addresses fear of the hospital. |
| | Demonstration of behaviour | Photographs/images help caregivers overcome fear, through becoming familiar with Queens. |
| Counselling | Prompt/cue | The booklet will be used as a tool for decision making about attending the referral, with the idea that it would be shared with caregivers/family members who did not attend the camp. |
| | Social support | Counsellors provide opportunity for two-way dialogue with people who understood the process. The expert mother has had a similar experience to those being referred and thus is able to share her personal experience. |
| | Information on health consequences | Counsellors discuss with caregivers the consequences of not attending the referral and answer key questions posed by caregivers. |
| Text message reminders | Prompt/cue | Text message reminders act as a prompt or cue for caregivers to remember to attend the referral once they had returned home from the screening camps. |


1. Michie, S., M. Richardson, M. Johnston, C. Abraham, J. Francis, W. Hardeman, M.P. Eccles, J. Cane, and C.E. Wood, *The behavior change technique taxonomy (v1) of 93 hierarchically clustered techniques: building an international consensus for the reporting of behavior change interventions.* Ann Behav Med, 2013. **46**(1): p. 81-95.DOI: 10.1007/s12160-013-9486-6.
