# Supplementary figures and images for: Developing Behaviour Change Interventions for Improving Access to Health and Hygiene for People with Disabilities: Two Case Studies from Nepal and Malawi
Source: Int J Environ Res Public Health. 2018 Dec 5;15(12):2746. doi: 10.3390/ijerph15122746 (PMC6313611; doi:10.3390/ijerph15122746)

# Theory of change for the menstrual hygiene behaviour change campaign

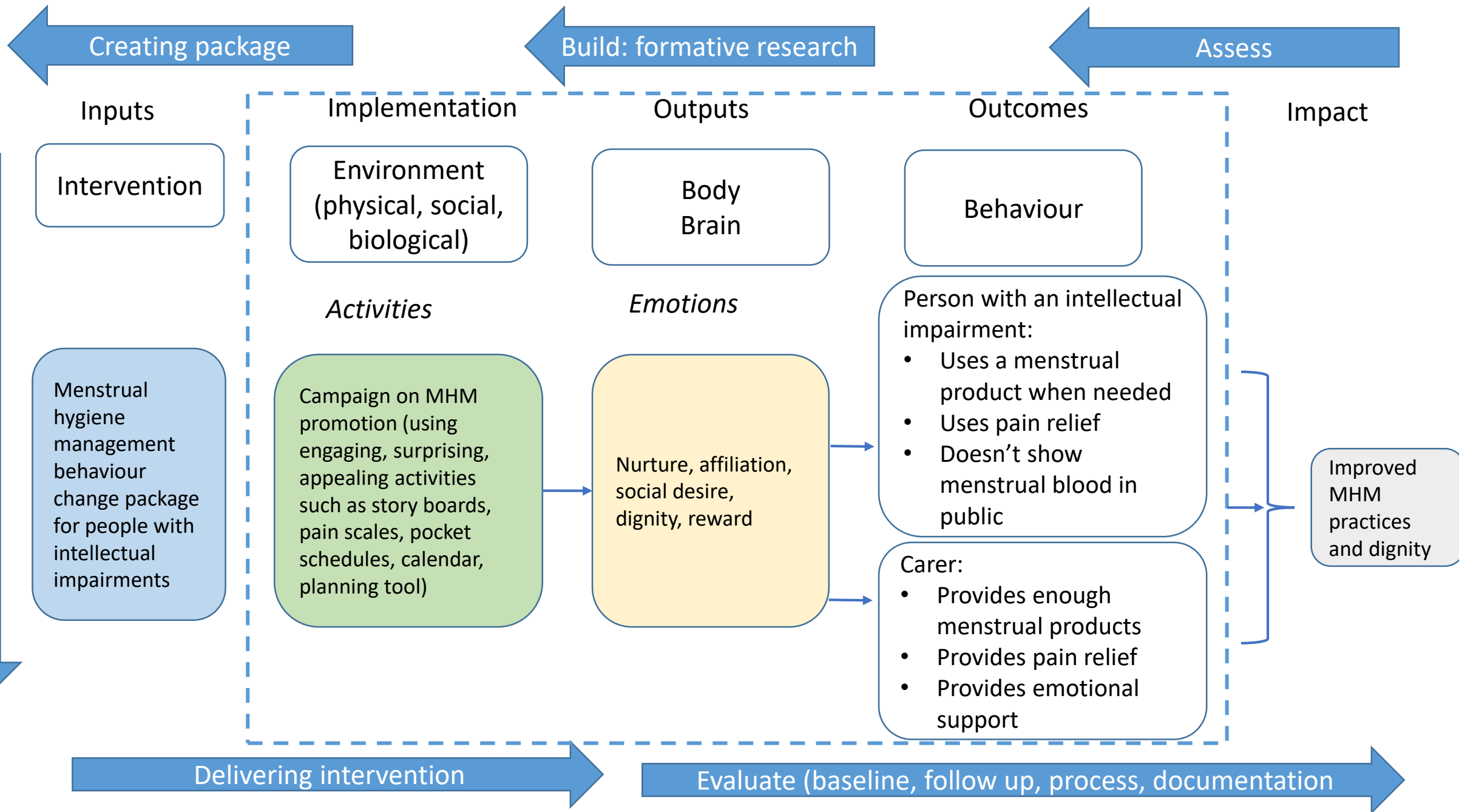

Supplement: Supplementary file 1 [file ijerph-15-02746-s001.zip › New folder/Figure_S2.pdf]
